# Bilateral cavernous sinus and left dural sigmoid sinus thrombosis associated with extreme exertion: a case report

**DOI:** 10.5935/0004-2749.20210013

**Published:** 2025-02-02

**Authors:** Chong Wern-Yih, Chan Jan-Bond, Menon Sudha, Abu Norlelawati, Ismail Shatriah

**Affiliations:** 1 Department of Ophthalmology, Hospital Tuanku Ja’afar, Negeri Sembilan, Malaysia; 2 Department of Ophthalmology, School of Medical Sciences, Universiti Sains Malaysia, Kerian, Kelantan, Malaysia

**Keywords:** Cavernous sinus thrombosis, Exercise, Physical training, Orbital cellulitis, Immunocompetence, Trombose do corpo cavernoso, Exercício físico, Celulite orbitária, Imunocompetência

## Abstract

Septic cavernous sinus thrombosis is a rare but often debilitating and potentially fatal disease. We describe a case of bilateral orbital cellulitis with rapidly progressing cavernous sinus thrombosis and left sigmoidal sinus thrombosis in an immunocompetent 20-year-old military man who had undergone intensive physical training. The patient presented with rapid painful swollen left eye for 2 days. The examination results were gross proptosis with total ophthalmoplegia. He was treated with intravenous antibiotics and corticosteroid. At 1 week, visual acuity improved to 20/20 OU, with a normal intraocular pressure. There was a significant improvement in proptosis. The ocular motility of the right eye was fully restored, with slight residual ophthalmoplegia in the left eye. There was no residual illness or recurrence of illness at 3 months’ follow-up.

## Abstract (pt)

A trombose séptica do seio cavernoso é uma condição rara, mas frequentemente debilitante e potencialmente fatal. Descrevemos um caso de celulite orbital bilateral com progressão rápida para trombose do seio cavernoso e trombose do seio sigmoide esquerdo, em um militar imunocompetente de 20 anos de idade que havia sido submetido a treinamento físico intenso. O paciente apresentou um inchaço rápido e doloroso no olho esquerdo por 2 dias. Os resultados do exame foram proptose macroscópica com oftalmoplegia total. Ele foi tratado com antibióticos intravenosos e costicosteróide. Em 1 semana, a acuidade visual melhorou para 20/20, com pressão intraocular normal. Houve uma melhora significativa na proptose. A motilidade ocular do olho direito foi totalmente restaurada, com leve oftalmoplegia residual no olho esquerdo. Não houve doença residual ou recorrência da doença após três meses de acompanhamento.

## INTRODUCTION

Cavernous sinus thrombosis (CST) has become an uncommon condition with the availability of antibiotics. The most common causes of CST are sinusitis, otitis, and odontogenic and facial skin infections^(1)^. In contrast to common belief, athletes and sportsmen are at increased risk of infections, especially during periods of heavy training^(2-3)^. We describe a case of rapidly progressing CST with left sigmoidal sinus involvement in an immunocompetent young man who had undergone intensive physical training.

## CASE REPORT

A 20-year-old healthy military man complained of a sudden, rapidly progressing painful and swollen left eye of 2 days’ duration. It was associated with blurring of vision, diplopia, and swelling of the left cheek. The patient had undergone intensive training in a military camp for 5 days, consisting of a total of 8 hours of running, marching, and physical strength training.

The patient was restless and febrile. The left maxillary area was swollen, tender, and warm. The neurological examination was normal except for a positive Kernig’s sign. Examinations of other systems were not suggestive of infection.

**Figure 1:**
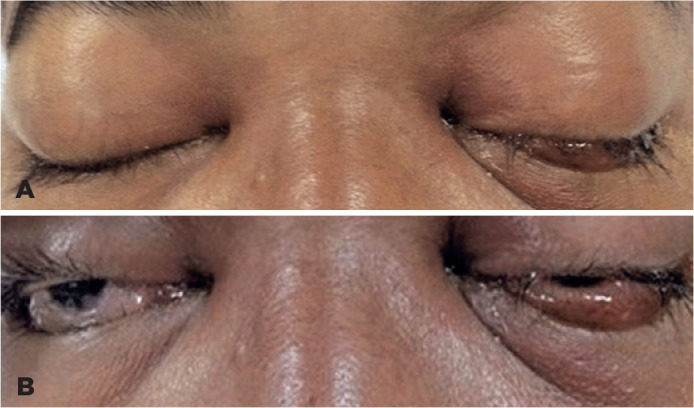
(A, B). Photographs on presentation showing bilateral gross proptosis and chemosis with left periorbital swelling.

Visual acuity was 20/30 OD and 20/60 OS. There was gross proptosis bilaterally (23 mm OD and 24 mm OS). There was total ophthalmoplegia with swollen and chemotic conjunctiva (Figure 1). The intraocular pressure was elevated (24 mmHg OD and 30 mmHg OS). No relative afferent pupillary defect was observed. The anterior and posterior segment examinations were normal.

The total white cell count was elevated to 23,000/µl, with predominantly neutrophils (86%). Blood culture was repeated twice and grew methicillin-sensitive *Staphylococcus aureus*. Lumbar puncture showed normal glucose and protein levels with negative culture results. Screening for thrombophilia, which included protein C, protein S, anti-thrombin III, activated protein C resistance, and lupus anticoagulant screening, was negative.

Contrast-enhanced computed tomography (CECT) showed bilateral prominent superior ophthalmic veins, proptosis, and frontal soft tissue swelling with an absence of loculation. However, no sign of distended cavernous sinus with a non-fat density-filling defect was observed.

**Figure 2:**
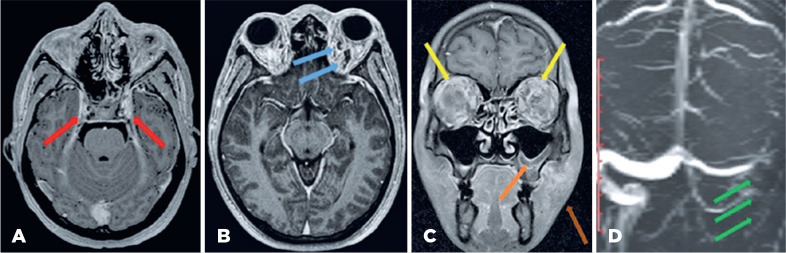
(A) Magnetic resonance imaging (MRI) showed features suggestive of bilateral cavernous sinus thrombosis (red arrow), (B) distended superior ophthalmic vein (blue arrow), (C) bilateral orbital cellulitis (yellow arrow), left maxillary sinusitis (orange arrow), and left cheek soft tissue swelling (brown arrow), (D) left sigmoid dural sinus thrombosis (green arrow).

Magnetic resonance imaging (MRI) revealed proptosis of both eyes with diffuse, symmetrical soft tissue thickening and areas of enhancement with engorgement of orbital vessels and inflammatory fat stranding involving the preseptal, postseptal, intraconal, and extraconal spaces. Magnetic resonance venography (MRV) did not show flow-related enhancement at the left sigmoid sinus. T2-weighted MRI showed a persistent filling defect within the cavernous sinus on both sides and the left sigmoid sinus. The sphenoid, left maxillary, and left ethmoidal sinuses showed mucosal thickening with an air-fluid level (Figure 2).

A diagnosis of bilateral orbital cellulitis and CST with evidence of left sigmoid sinus thrombosis complicated by sphenoid, left ethmoidal, and maxillary sinusitis was made. The patient was started on IV ceftriaxone 2 g twice daily, IV cloxacillin 1 g 6 hourly, IV hydrocortisone 50 mg 8 hourly, and IV heparin 6000 IU 6 hourly. Gutta moxifloxacin 4 hourly, gutta timolol 12 hourly, and gutta hydroxypropylmethylcellulose 6 hourly were prescribed for both eyes.

**Figure 3:**
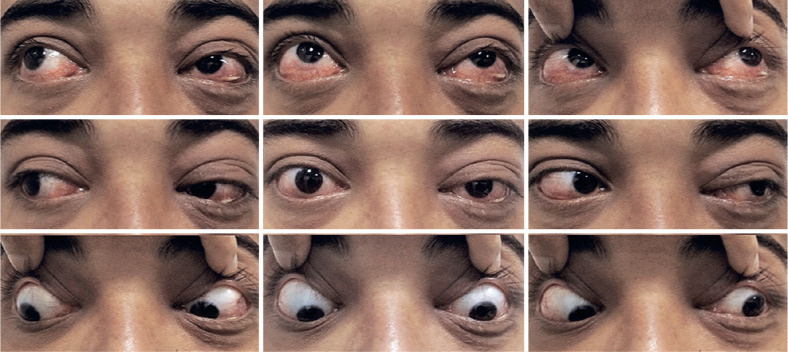
Photographs after completion of treatment. Nine-gaze photographs showed resolution of proptosis and ophthalmoplegia.

At 1-week, visual acuity improved to 20/20 OU, with a normal level of intraocular pressure. There was a significant improvement in proptosis. Ocular motility of the right eye was fully restored, with slight residual ophthalmoplegia in the left eye (Figure 3). There was no residual illness or recurrence of illness at 3 months’ follow-up.

## DISCUSSION

CST is the rarest form of dural venous sinus thrombosis, with a mortality rate of up to 30% and a morbidity rate as high as 22%^(2)^. The most common causes are sinusitis, otitis, and odontogenic and facial skin infections^(1)^ due to the close proximity of the structures^(3)^. Our case report showed an increased risk of infection in a healthy individual due to extreme physical exertion.

It has been reported that intensive training and overtraining are associated with immunodepression and susceptibility to infection^(2,3)^. The “inverted J hypothesis” in exercise immunology suggested that disease susceptibility increased in sedentary and over-trained subjects in comparison with subjects who underwent regulated, moderate training. Depression of natural killer cell function, reduction in expression of toll-like receptors, and increased release of cortisol and proinflammatory cytokines, such as tumor necrosis factor, interleukin-1b, and interleukin-6, are well-documented mechanisms that may have contributed to the immunocompromised state of our patient^(4)^.

Another possible mechanism of the rapid progression of CST to intracranial thrombosis is the rebalanced hemostatic state, simultaneously causing hypercoagulability and enhanced fibrinolysis induced by extreme physical exertion. Hypercoagulability appears to persist longer, from a few hours to a day, than fibrinolytic activities after strenuous training^(5)^. Hence, we presumed that our patient was likely in a prothrombic state, which favored intracranial thrombus formation.

*Staphylococcus aureus* is the most common organism isolated in CST. Other organisms, including *Streptococcus*, fungi, Enterobacteriaceae, and anaerobes, have also been reported^(6)^. Predisposed to an increased risk of in fection associated with extreme physical exertion^(4)^, our patient developed methicillin-sensitive *S. aureus* septicemia from uncomplicated sinusitis and progressed to bilateral CST with left sigmoid sinus involvement. Our case is similar to the cases described by van der Poel et al., who reported that most of their patients recovered without any permanent deficits^(7)^.

High-resolution CECT provides superb bone-air soft tissue details of the orbit and sinuses. CST may present as multiple irregular filling defects in the cavernous sinus on CECT. Our patient had normal CECT scan findings, similar to the report of Komatsu et al.^(8)^. Thus, the managing clinician should have a high index of suspicion towards the diagnosis of CST based on clinical presentation, even if the CECT scan is normal.

High-resolution MRI visualizes the enlargement of the CST as filling defects over time. Thin-slice gradient recalled echo sequences with gadolinium contrast have been shown to be more sensitive than routine MRI pulse sequences for detection of filling defects in the cavernous sinus^(9)^. MRV is helpful when the clinician suspects that dural sinuses are involved. In our case, MRI and MRV mapped out the complete extension of the CST in detail.

CST remains a life-threatening condition. Our case is an example of intensive physical training as a possible cause of thrombosis in a young patient with a rapid onset of intracranial thrombosis.
